# “RaMassays”: Synergistic Enhancement of Plasmon-Free Raman Scattering and Mass Spectrometry for Multimodal Analysis of Small Molecules

**DOI:** 10.1038/srep34521

**Published:** 2016-10-04

**Authors:** Ivano Alessandri, Irene Vassalini, Michela Bertuzzi, Nicolò Bontempi, Maurizio Memo, Alessandra Gianoncelli

**Affiliations:** 1 INSTM and Chemistry for Technologies Laboratory, Mechanical and Industrial Engineering Department (DIMI), University of Brescia, via Branze 38, 25123 Brescia, Italy; 2 INSTM and Department of Molecular and Translational Medicine, University of Brescia, Viale Europa 11, 25123, Brescia, Italy

## Abstract

SiO_2_/TiO_2_ core/shell (T-rex) beads were exploited as “all-in-one” building-block materials to create analytical assays that combine plasmon-free surface enhanced Raman scattering (SERS) and surface assisted laser desorption/ionization (SALDI) mass spectrometry (RaMassays). Such a multi-modal approach relies on the unique optical properties of T-rex beads, which are able to harvest and manage light in both UV and Vis range, making ionization and Raman scattering more efficient. RaMassays were successfully applied to the detection of small (molecular weight, M.W. <400 Da) molecules with a key relevance in biochemistry and pharmaceutical analysis. Caffeine and cocaine were utilized as molecular probes to test the combined SERS/SALDI response of RaMassays, showing excellent sensitivity and reproducibility. The differentiation between amphetamine/ephedrine and theophylline/theobromine couples demonstrated the synergistic reciprocal reinforcement of SERS and SALDI. Finally, the conversion of L-tyrosine in L-DOPA was utilized to probe RaMassays as analytical tools for characterizing reaction intermediates without introducing any spurious effects. RaMassays exhibit important advantages over plasmonic nanoparticles in terms of reproducibility, absence of interference and potential integration in multiplexed devices.

Matrix-Assisted Laser Desorption/Ionization mass spectrometry (MALDI/MS) and Surface Enhanced Raman Scattering (SERS) have been growing at a fast pace, achieving impressive breakthroughs in advanced diagnostics^1^,^2^,^3^,^4^,^5^. Their synergistic combination could open exciting opportunities in pharmacology and biochemistry (drug design, xenobiotics, “-omic sciences”, including metabolomics, peptodomics, glycomics, lipidomics, methylomics.) forensic and environmental chemistry, tissue and cell analysis etc^6^,^7^,^8^,^9^,^10^.

However, the development of multi-modal detection tools is currently hampered by several critical drawbacks, affecting both techniques. Conventional MALDI/MS relies on the ionization of organic matrices (2,5-dihydroxy benzoic acid, α-cyano-4-hydroxycinnamic acid, picolinic acid and related compounds, isovanillin.), which introduces high background signals in the low-mass region (<400 Da). As a result, MALDI/MS detection is made difficult or even prevented for a number of small molecules that play a key role in the above-mentioned research topics (neurotransmitters, hormones, metabolites, toxins, pharmaceutical and illicit drugs, pigments, explosives, environmental pollutants). Traditional organic matrices are also often characterized by low reproducibility, as a consequence of inhomogeneous crystallization of the analytes^11^,^12^.

These limitations have been partly overcome by using nanoparticles and nanostructures as matrices. This approach, which is also referred as surface assisted laser desorption/ionization (SALDI/MS), exploits the confinement of electrons, excitons, plasmons and/or phonons, associated to the reduced dimension of the nanostructured matrix, to promote ionization^13^,^14^,^15^,^16^,^17^.

However, MALDI/MS is not sufficient to extract direct structural information on small molecules at low concentration. In this regard, vibrational spectroscopy may complement mass analysis, providing the chemical fingerprint of any analyte. In particular, because of its relative insensitivity to water, Raman microspectroscopy is a powerful tool for characterizing small molecules in their natural aqueous working environment. Plasmon-assisted SERS has been extensively investigated in order to obtain high sensitivity in molecular detection. However, the strong enhancement of the local electromagnetic field resulting from interaction of the laser source with plasmonic metals and related thermal dissipation can severely perturb the molecular systems under analysis, especially when dynamic processes are investigated. For this reason, most the present research on SERS-active substrates has been focused on all-dielectric materials, which offer many advantages in terms of low-invasiveness, reproducibility and manifold opportunities to excite different optical resonances^18^,^19^,^20^,^21^. Among those, TiO_2_, C/TiO_2_ composites and SiO_2_/TiO_2_ core/shell (T-rex) beads demonstrated excellent sensitivity towards different kind of molecules (environmental pollutants, amino acids, small peptides…), allowing for plasmon-free SERS detection under real working conditions^22^,^23^,^24^,^25^,^26^,^27^,^28^. In the case of T-rex beads, their SERS activity is based on their light-trapping capability, which can be controlled by trimming the thickness of the titania layer in single beads, as well as the number of beads assembled in three-dimensional colloidal crystals^26^,^27^,^28^. The TiO_2_ surface can be covalently functionalized with appropriate receptors^29^ that impart the selectivity needed for detecting specific molecules in the presence of either similar or interfering molecular species^30^. As a further benefit, the photocatalytic activity of TiO_2_ irradiated by UV-light can be exploited for self-cleaning and re-using the same substrate for further analysis, which is crucial for achieving spot-to-spot reproducibility and direct comparison among different reactions occurring exactly at the same place^26^,^27^,^28^,^31^,^32^. UV light absorption of nanostructured titania can be also exploited to enhance the efficiency of desorption/ionization processes in SALDI/MS, which typically utilizes ionization sources based on UV lasers (*e.g.* N_2_ lasers: λ = 337 nm)^33^.

Until now there are only few reports on SERS-SALDI/MS coupled assays, and all of them have been limited to plasmonic nanoparticles (Au, Ag)^7^,^8^,^9^,^10^. However, in most of the cases those nanoparticles are stabilized using surfactants or other capping agents that can interfere with the detection of low molecular mass analytes^34^,^35^. Other sources of interference might result from the production of ionized clusters, as reported in the case of gold nanofilms^36^. Moreover, optimal conditions for SERS do not match those necessary for an optimal SALDI/MS response. T-rex beads could bridge this gap enabling “all-in-one” plasmon-free SERS and organic matrix-free mass analysis. This synergistic merging relies on the fact that the efficiency of both UV light-induced ionization and Visible light trapping-induced SERS depend on the thickness of the titania shell, which in T-rex can be easily and accurately controlled by means of atomic layer deposition (ALD). This approach introduces the concept of T-rex-based “RaMassays” (Raman + Mass assays), which is demonstrated here in detection of a series of small molecules (caffeine and related xanthines, illicit drugs, like cocaine and amphetamine, neurotransmitters and their precursors) with molecular mass in the range 100–400 Da, as well as through experiments in which the synergistic combination of both techniques is necessary to obtain unambiguous results.

## Results

### RaMassays: proof-of-concept

**Figure 1:**
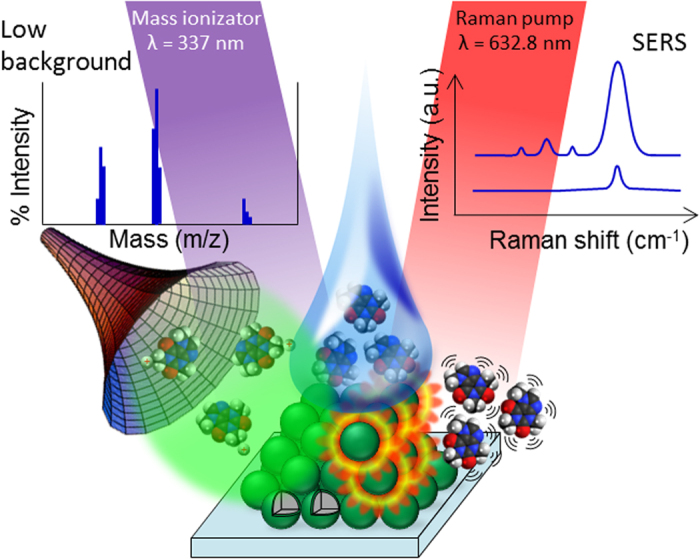
The RaMassay concept. 3D T-rex colloidal crystals are exploited as a highly efficient, background-free matrix for mass analysis and all-dielectric substrate for plasmon-free SERS.

The RaMassays were based on T-rex beads, fabricated according to a procedure reported in previous papers^26^,^32^. In the present case the thickness of the anatase shell layer, obtained through conformal ALD, was set at 100 nm, which ensures good efficiency in both SERS and laser desorption/ionization processes. Three-dimensional crystals of T-rex beads were obtained by self-assembly upon room temperature sedimentation of aqueous suspensions (*see* Supporting Information SI 1). These colloidal crystals served as substrates for both SERS and mass analysis. Further details on the experimental setup are reported in the Methods section. As already demonstrated in previous works^26^,^27^,^28^,^30^, the supra-crystals obtained from T-rex beads play a twofold role in SERS detection: they are very efficient in trapping the incoming light from the laser exciting source, extending the optical path-length through multiple scattering and internal reflection. In addition, they allow the analyte dissolved in highly diluted solutions to be pre-concentrated at the substrate surface. These key features offer major advantages in terms of direct detection of analytes from solutions, avoiding extra-functionalization with labels or signal reporters. In addition, different Raman experiments can be carried out on specific beads that can be selected by direct optical microscope inspection, which increases reproducibility and comparability of data. At the same time, as a result of the reduced thickness of TiO_2_ shell, which is commensurate with the wavelength of the UV laser utilized for laser/desorption ionization in commercial MALDI/TOF instruments (λ = 337 nm), T-rex beads are expected to promote the analyte ionization and acquisition of mass spectra unaffected by the high background which is typically associated to organic matrices (*see* Scheme in Fig. 1).

**Figure 2:**
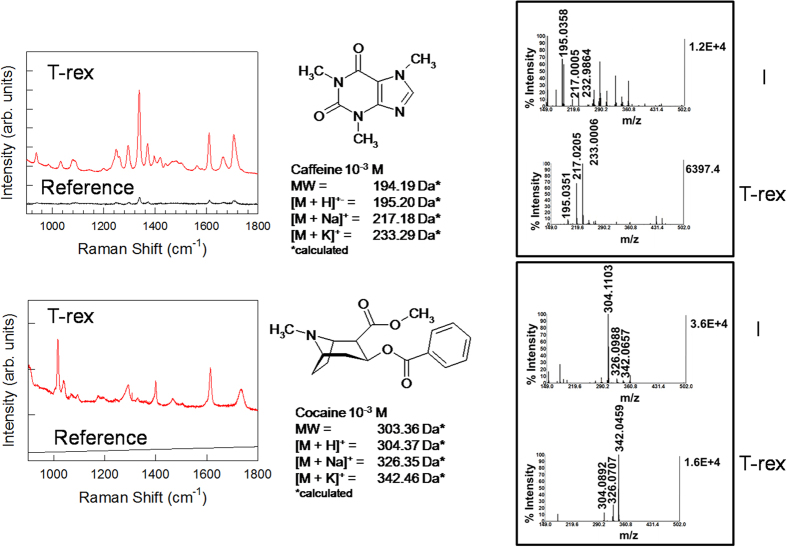
RaMassay at work: Raman and mass detection of caffeine and cocaine solutions. Examples of combined plasmon-free SERS (left) and SALDI/MS (right) detection of small molecules, caffeine (M. W.: 194.19 Da, on the top) and cocaine (M.W.: 303.36 Da, on the bottom). The Raman spectra acquired with T-rex beads are compared to reference spectra from analyte solutions with the same concentration (10^−3^ M) deposited on a glass microscope slide. The SALDI/MS spectra acquired with T-rex beads are compared with those acquired using a conventional organic matrix, isovanillin, indicated by I.

We tested the concept of “RaMassay” using 3D T-rex supracrystals in combined dual-mode (SERS and mass) detection of caffeine (M.W.: 194.19 g/mol) and cocaine (M.W.: 303.35 g/mol), chosen to represent paradigms of very important psychoactive alkaloids with molecular weight below 400 Da (Fig. 2).

The Raman enhancement obtained from T-rex beads clearly emerges from a direct comparison with the reference spectra acquired under the same conditions from analyte solutions deposited onto glass slides. For example, the caffeine recrystallized on the T-rex arrays from (10^−3^M) solutions clearly exhibits the typical Raman peaks of C-N, C=N, C=O and N-CH_3_ stretching modes^37^.

On the contrary, the intensity of the Raman spectra of caffeine recrystallized from reference solutions at the same concentration is very weak (I ratio ∼1/14 at 1610 cm^−1^).

T-rex beads also promoted detection of cocaine, resulting in enhanced Raman spectra showing the characteristic modes of O-C=O ester stretching (∼1280 cm^−1^) and C = O stretching of the benzoate ester (∼1724 cm^−1^), as well as the very strong breathing (∼1000 cm^−1^) and stretching (∼1600 cm^−1^) modes of the aromatic ring^38^.

In the case of cocaine and many other analytes investigated in the present (tyrosine, L-DOPA, amphetamine, ephedrine) or previous (glutathione)^28^ studies, the concentrations of reference solutions (10^−3 ^M) are below the detection limit. In this respect, T-rex beads are essential not only to enhance the Raman response, but also to make non-resonant analytes detectable below the millimolar range without introducing any molecular labels or SERS reporters. Moreover, as already demonstrated in previous works^26^,^27^, the Raman spectra on 3D T-rex supracrystals are characterized by high reproducibility (relative standard deviation, R.S.D. <10%).

The combined mass spectrometry data revealed the key advantages of using T-rex instead of conventional matrix for small molecules, like isovanillin. As shown in the examples, the presence of the organic matrix (indicated by “I” in Fig. 2) overwhelms the signals originating from the analytes, with detrimental effects on spectral readout. On the other hand, T-rex offers remarkably better performances in terms of ionization efficiency and overall sensitivity. The absence of spurious contributions from the organic matrix allows for the unambiguous collection of mass signals exclusively originating from the analytes. Moreover, no preliminary investigation on chemical compatibility between an organic matrix and the analyte is needed.

Further analysis on caffeine revealed that the limit of detection for both SERS and mass spectrometry was set around same concentration (10^−5^–10^−6 ^M). (SI 2) In general, this range of concentrations corresponds to that of physiological interest for small molecules with biological activities, like drugs or neurotransmitters^39^. Analogous results were found for the other molecules investigated in the present work.

T-rex beads are also effective in detecting analytes in the presence of phosphate buffers over a wide range concentration (0.5–100 mM). In general salts of phosphate buffer components affect the sensitivity of a MALDI analysis by quenching ionization^40^.

This holds true also for T-rex, causing an overall reduction of the intensity of the mass signals. However, the low-background of TiO_2_ allows sensitivity to be maintained even for 100 mM phosphate buffer solutions, that is a quite high concentration in comparison to that utilized in experiments involving biological-active molecules (5–20 mM, *see* SI 3).

### Mutual Assistance

**Figure 3:**
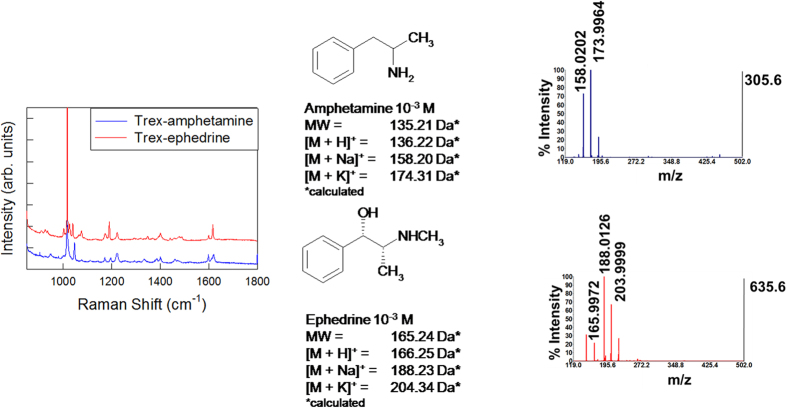
SERS-SALDI/MS mutual assistance: SALDI disambiguation of structurally -related drugs. Example of RaMassay that takes advantage of SALDI/MS to discriminate between amphetamine (blue SERS and SALDI/MS spectra) and ephedrine (red SERS and SALDI/MS spectra, both 10^−3^ M solutions), two structurally-related drugs with similar Raman spectra but different molecular weight.

The combined use of SERS and mass spectrometry offers important advantages whenever the individual application of either technique yields ambiguous results. For example, Raman spectra of structure-related compounds are often very similar. Very specific receptors and/or time-consuming data analysis are required for a clear identification. However, the use of mass spectrometry could make detection direct and very simple. Figure 3 shows the Raman spectra of (1R, 2S)-(-)-ephedrine and amphetamine, two important alkaloids related by the same phenethylamine skeletal structure. Both spectra display almost the same modes, which are related to the benzene ring breathing vibrations, the only difference being their relative intensity^41^,^42^.

In this case a reliable differentiation between the two analytes cannot be obtained at glance, asking for an accurate analysis accounting for any possible pitfalls related to crystallization from solutions. However, since the two molecules have a different molecular weight, the matrix-free mass analysis provides an immediate support to identification.

**Figure 4:**
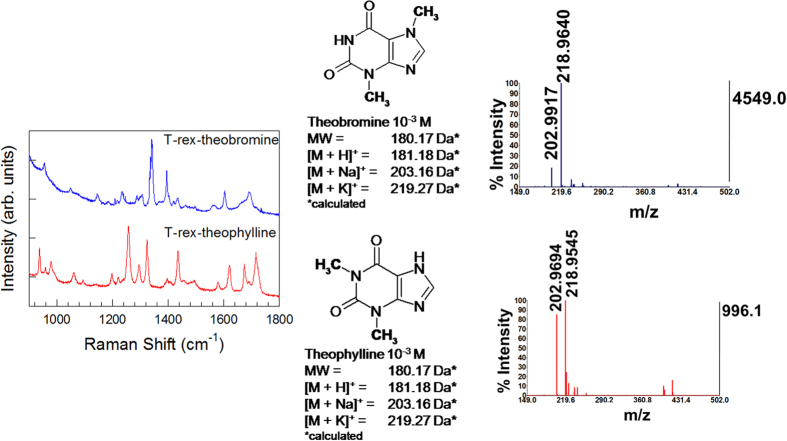
SERS-SALDI/MS mutual assistance: SERS detection of two regioisomers. Example of RaMassay that takes advantage of plasmon-free SERS to discriminate between theobromine (blue SERS and SALDI/MS spectra) and theophylline (red SERS and SALDI/MS spectra, both 10^−3^ M solutions), two caffeine metabolites which have the same molecular weight (regioisomers) and, therefore, cannot be discriminated by SALDI/MS.

On the other hand, in the case of regioisomers, Raman analysis is a decisive tool for identifying different compounds. An example is shown in Fig. 4. Here theophylline and theobromine, two dimethylxantines produced in metabolic degradation of caffeine, having the same molecular formula (C_7_H_8_N_4_O_2_, MW: 180.17 g/mol) yet different position of methyl-groups attached to the xanthine ring, were unambiguously identified with the aid of Raman analysis^37^.

The cases of either regioisomers or compounds containing moieties dominating the overall Raman spectrum represent examples of mutual compensation, in which one technique addresses the flaws of the other one. However, as demonstrated in experiments with caffeine and cocaine, in most of the cases both Raman and mass spectrometry play together to provide extensive chemical information on the analytes.

### Analysis of reaction intermediates/products without metal interference

**Figure 5:**
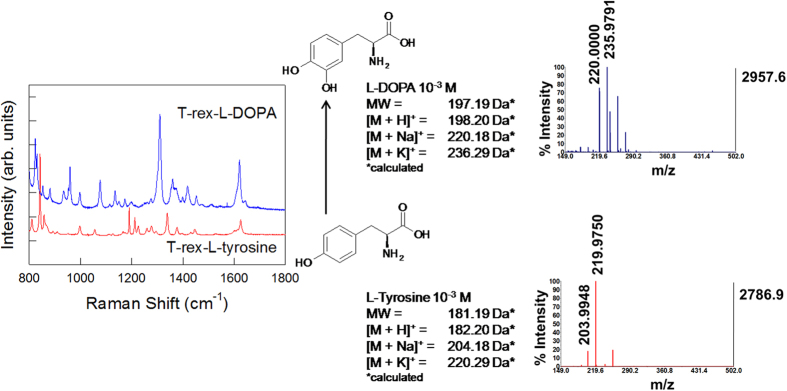
Analysis of reaction intermediates/products without metal interference. Ramassay utilized to detect L-tyrosine and L-DOPA 10^−3^ M solutions. The addition of a second OH- group improves the anchorage on T-rex and promotes charge-transfer between the target molecule and TiO_2_ shell, which determines a strong enhancement of the Raman spectrum of L-DOPA (blue spectrum) in comparison to L-tyrosine (red spectrum). This effect, combined to the precise SALDI/MS (right) identification of the molecular targets can be exploited for monitoring intermediates and products of various chemical reactions in solution without the interference of any metallic enhancers.

Another key aspect of RaMassays is their potential use in characterizing reaction intermediates and products with great details. Figure 5 shows the combined analysis of L-tyrosine and L-DOPA. In dopaminergic cells L-tyrosine is converted to L-DOPA through the addition of a second hydroxyl group to the benzene ring by means of tyrosine hydroxylases. Those closely related molecules can be distinctively detected using RaMassays. Here it is also interesting to note that the addition of the second hydroxyl allows L-DOPA to be strongly attached to the TiO_2_ surface through a bidentate bridge. As already reported by Rajh and co-workers in their seminal studies on SERS effect in titania nanoparticles^43^, this particular coordination enables the formation of an efficient charge-transfer complex, which strongly contributes to the overall Raman enhancement. The simple removal of one of these hydroxyl groups is sufficient to weaken the Raman response. Thus, the formation of L-DOPA can be selectively highlighted by a remarkable increase of the Raman intensity, which is useful in view of multiplexed analysis.

## Conclusion and Outlook

In summary, this study introduced the concept of RaMassays as new multi-modal tools for achieving plasmon-free SERS and matrix-free mass analysis. This approach overcomes the limitations of both conventional SERS and MALDI, allowing for sensitive and reliable detection of small (M.W. < 400 Da) molecules that play important biological functions, like illicit drugs and neurotransmitters. The main advantages are related to the absence of any spurious molecules, the possibility to monitor chemical reactions with precise spatial localization with both mass and vibrational spectroscopy and without metal-induced interferences, reproducibility and full recyclability of the materials, integration in commercial devices (*e.g.* MALDI/TOF plates), potential for multiplexing and combined Raman/mass imaging^44^.

Future challenges will be addressed to a deeper understanding of the laser desorption/ionization and light trapping mechanisms, in order to provide a complete guideline to the design of a second-generation of multi-modal analytical platforms, with new functionalities extended to separation techniques and infrared spectroscopy^45^. Another open issue is represented by the possible extension of RaMassays to ultra-trace detection of explosives and micropollutants, which has been already reached by plasmon-assisted SERS^46^ or hybrid metal/dielectric compounds^47^,^48^,^49^,^50^,^51^,^52^, but still needs to be coupled with mass spectrometry. In this regard, further improvements in sensitivity might be obtained by integrating T-rex beads and related composites, made of shell oxide-protected metals and high-refractive index dielectrics, into light-trapping architectures^53^.

## Methods

### Materials

All the analytes (caffeine, theobromine, theophylline, L-DOPA, L-tyrosine, cocaine hydrochloride, amphethamine, 1R, 2S-(-)-ephedrine hydrochloride solution (1mg/mL in methanol)), isovanillin, one of the matrix for MALDI-TOF/TOF–MS analysis, acetonitrile (98% purity, CH_3_CN) and trifluoroacetic acid (99% purity, TFA), *i.e.* the solvents for matrix preparation, as well as sodium phosphate monobasic (NaH_2_PO_4_) salt utilized for buffered samples were provided from Sigma-Aldrich.

### Preparation of T-rex beads

The synthesis and characterization of T-rex beads followed the protocols described in previous papers^26^,^32^. Briefly, monodispersed SiO_2_ spheres (diameter: 2 μm) were coated with a conformal, 100 nm-thick layer of TiO_2_ by ALD from tetrakis(dimethylamino)titanium(IV), 99.99% (Sigma-Aldrich) precursor. Post-deposition annealing in air at 700 °C transformed amorphous TiO_2_ into crystalline anatase.

### Raman Analysis

The microRaman analysis was carried out in backscattering configuration using a Labram HR-800 (Horiba-Jobin Yvon) equipped with an optical microscope (BX41; Olympus Optical Co. Ltd.). All the spectra were acquired using a 632.81 nm HeNe laser source with a 100X (Numerical Aperture, N.A.: 0.9) microscope objective and high-resolution grating (1800 grooves/mm). 10 different regions of T-rex crystals were sampled in each Raman experiment and 3 spectra per region were acquired (30 spectra for each analyte).

### Matrix-Assisted Laser Desorption/Ionization Mass Spectrometry [MALDI-TOF/TOF–MS] Analyses

MALDI-TOF/TOF analyses were carried out with an AB SCIEX TOF/TOF MS 5800 System (Sciex, Framingham, MA, USA), equipped with a nitrogen laser (λ = 337 nm). Measurements were performed using the instrument in reflector positive mode. Spectra were recorded in a mass range from 150 to 500 Da with a focus mass of 200 Da. All mass spectra resulted from accumulation of at least 1500 laser shots using a random search pattern. The laser intensity was setting at 5000 with a pulse laser of 400 Hz for all analyses, and the detector voltage multiplier was fixed at 0.52.

### T-rex versus isovanillin

The two different matrices compared in the present work were prepared in the following way: a) 10 mg of isovanillin were dissolved in 1 mL of 70:30 CH_3_CN: TFA0.1%, b) 1 mg of T-rex beads were scratched from the substrate, dispersed in 100 μL of milliQ-grade water (10 mg/mL solution) and sonicated (UST/41, 38 KHz) for 15 minutes.

The stock solutions of all the analytes were prepared by dissolving the powders or original solutions in milli-Q water to obtain final concentration of 10^−3^ M.

To evaluate the usefulness of T-rex beads as matrix for identification of small molecules in a MALDI-TOF/TOF–MS analysis in comparison to the organic matrix isovanillin, each molecule was analyzed with both matrices. The “thin layer method” was used as sample-matrix deposition procedure^45^. For each compound, 1 μL of each matrix, T-rex or isovanillin, was deposited on the arrayed wells of a 384 Opti TOF 123 × 81 mm target plate and allowed to dry, then 1 μL of each target solution was applied on matrices and allowed to dry.

### Limit of detection in MALDI-TOF/TOF MS analysis

Caffeine was utilized to evaluate the limit of detection both for MALDI-TOF/TOF MS in the presence of both matrices. For MALDI-TOF/TOF MS analysis, different concentrations (10^−4^ M, 10^−5^ M, 10^−6^ M) of caffeine solution in milli-Q water were prepared by serial dilution started from the 10^−3^ M stock solution and analyzed with both matrices.

The analysis of the solutions at different concentrations was carried out using the “thin layer method” described above.

### Salt interferences in MALDI-TOF/TOF MS analysis

Caffeine solutions at concentration of 10^−3^ M were prepared dissolving caffeine powder in phosphate buffer at different concentration (0.5 mM, 1 mM, 5 mM, 10 mM, 20 mM, 50 mM and 100 mM). Also in this case, each solution was analysed by MALDI-TOF/TOF MS using both T-rex beads and isovanillin matrices, using the same protocol described before. The results for three different buffer concentrations (0.5, 5 and 50 mM) were reported in SI 3.

## Additional Information

**How to cite this article**: Alessandri, I. *et al*. ‘‘RaMassays’’: Synergistic Enhancement of Plasmon-Free Raman Scattering and Mass Spectrometry for Multimodal Analysis of Small Molecules. *Sci. Rep.* **6**, 34521; doi: 10.1038/srep34521 (2016).

## Supplementary Material

Supplementary Information
