# Supplementary material for: “RaMassays”: Synergistic Enhancement of Plasmon-Free Raman Scattering and Mass Spectrometry for Multimodal Analysis of Small Molecules
Source: Sci Rep. 2016 Oct 4;6:34521. doi: 10.1038/srep34521 (PMC5048303; doi:10.1038/srep34521)
Supplement: Supplementary Information [file srep34521-s1.pdf]

“RaMassays”: Synergistic Enhancement of Plasmon-Free Raman Scattering and Mass Spectrometry for Multimodal Analysis of Small Molecules

Ivano Alessandri,[a]* Irene Vassalini,[a] Michela Bertuzzi,[b] Nicolò Bontempi,[a] Maurizio Memo,[b] Alessandra Gianoncelli[b]*

[a] INSTM and Chemistry for Technologies Laboratory, Mechanical and Industrial Engineering Department (DIMI), University of Brescia, via Branze 38, 25123 Brescia, Italy.

[b] INSTM and Department of Molecular and Translational Medicine, University of Brescia, Viale Europa 11, 25123, Brescia, Italy.

* corresponding authors:

e-mail: ivano.alessandri@unibs.it

alessandra.gianoncelli@unibs.it

SUPPORTING INFORMATION

S1. SEM image of T-rex beads utilized as RaMassays.

S2 A-D. Investigation of the limit of detection (Raman and SALDI/MS) for the caffeine RaMassay.

S3. A-C. Investigation of the SALDI/MS response of the caffeine RaMassay (caffeine solution: 10^{-3} M) in phosphate buffer solutions (0.5-50 mM).

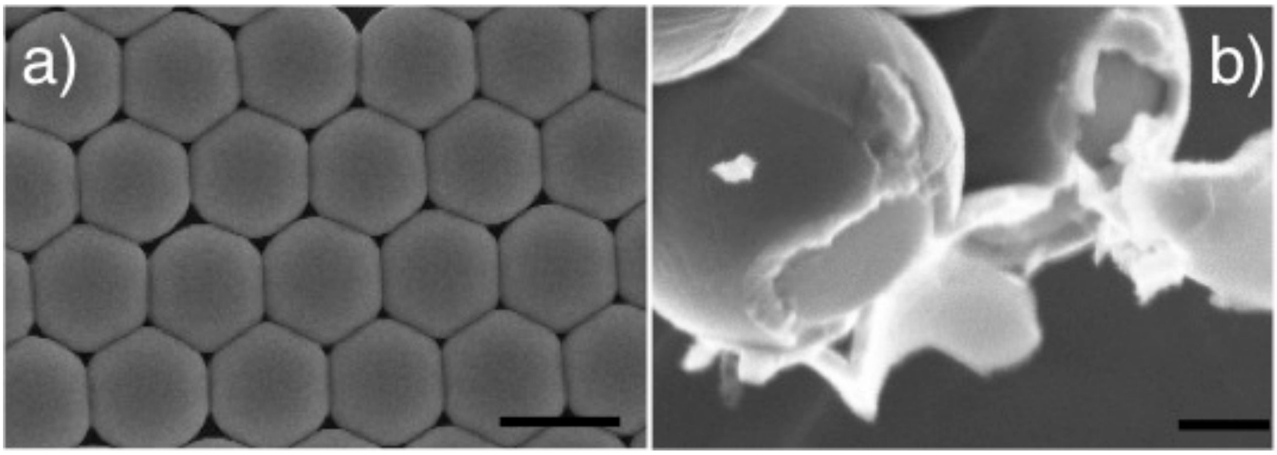

S1. SEM image of T-rex beads utilized for RaMassays: a) top view image of a 3D assembly (scale bar: 2 μm); b) crushed specimens showing a cross-sectional view of the core/shell microstructure made of SiO_2 spherical cores and TiO_2 shell layers (scale bar: 500 nm). Further details on structural characterization of T-rex beads can be found in refs. 26 and 32.

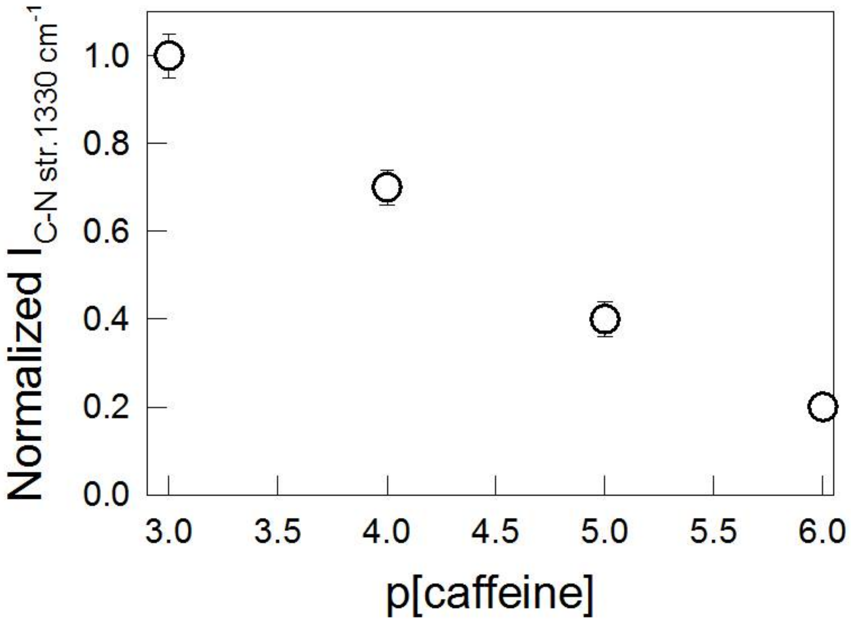

S2-A. Normalized intensity of the C-N stretching mode at about 1330 cm^{-1}, taken as a reference to evaluate the detection limit for caffeine solutions at different concentrations (p[caffeine]=-log[caffeine]). The limit of detection was 10^{-6} M (for 10^{-7} M solution the S/N ratio is <3). Each point refers to the mean intensity values acquired from 30 spectra.

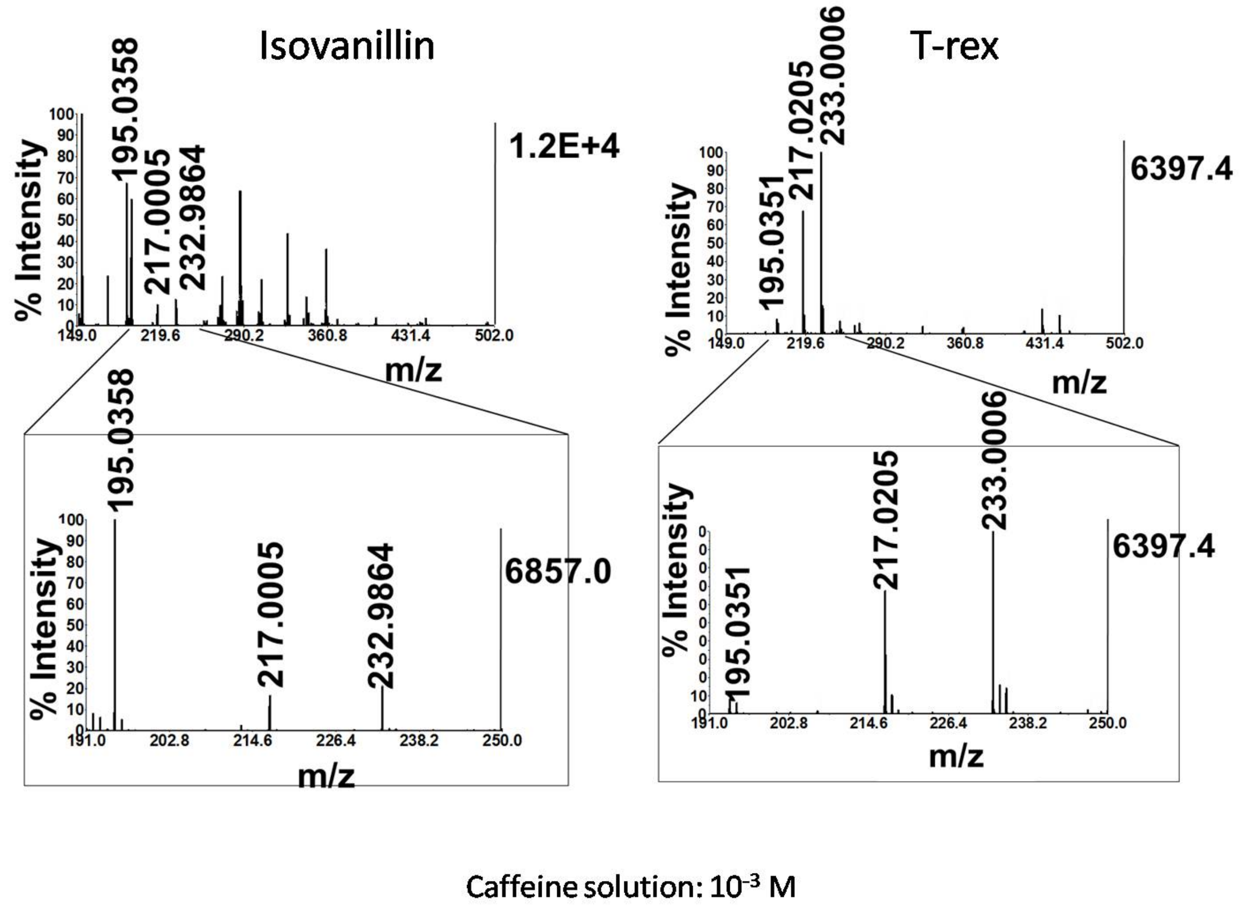

S2-B. Comparative evaluation of SALDI/MS detection limit for caffeine RaMassays and isovanillin matrix (caffeine solution concentration: 10^{-3} M).

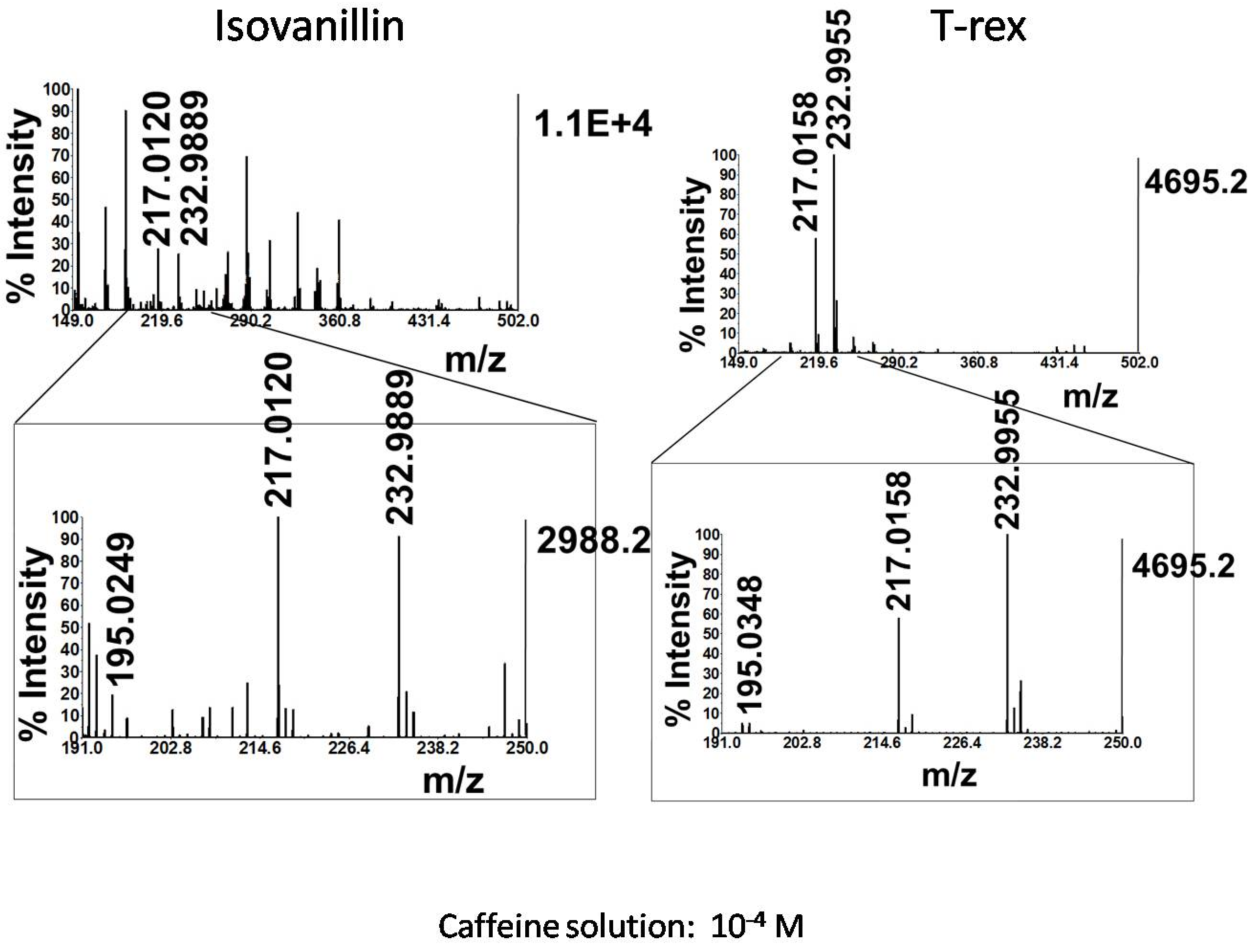

S2-C. Comparative evaluation of SALDI/MS detection limit for caffeine RaMassays and isovanillin matrix (caffeine solution concentration: 10^{-4} M).

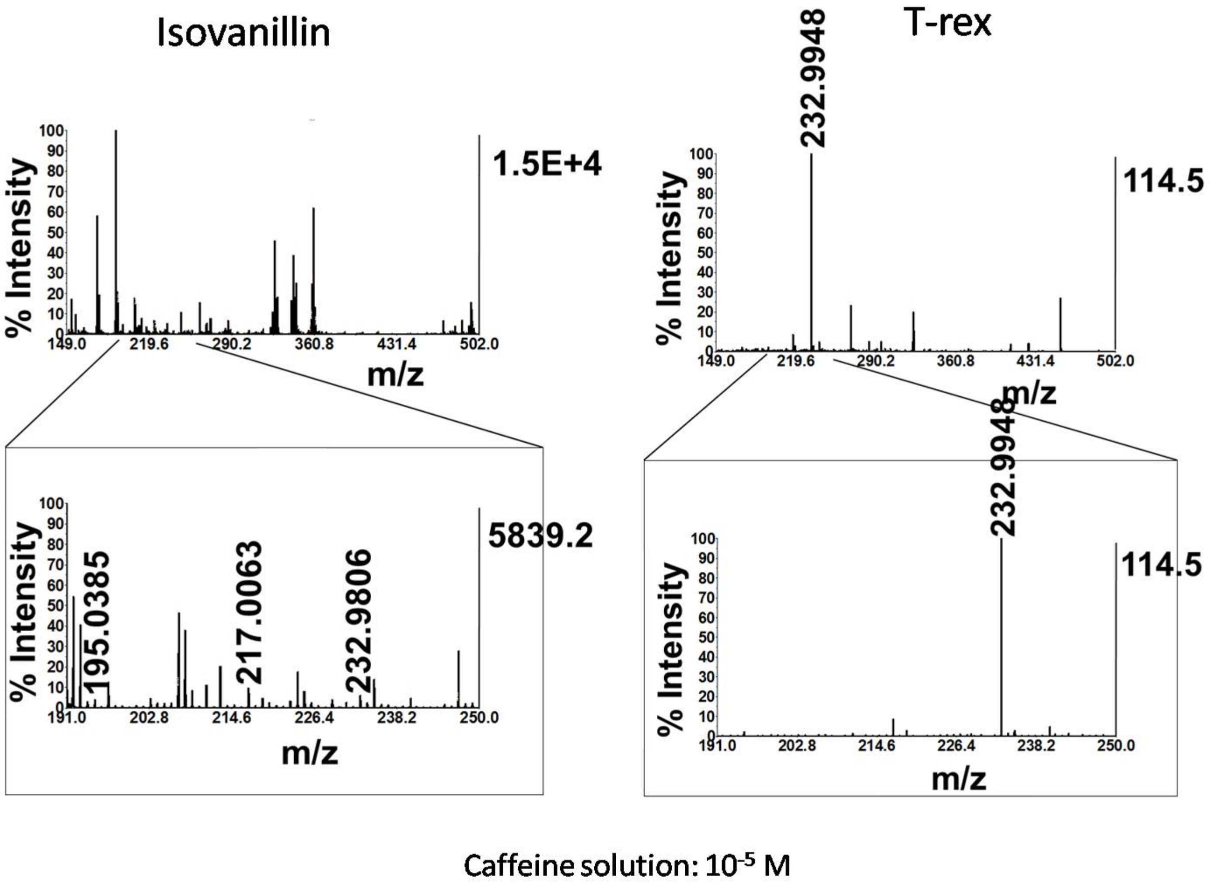

S2-D. Comparative evaluation of SALDI/MS detection limit for caffeine RaMassays and isovanillin matrix (caffeine solution concentration: 10^{-5} M).

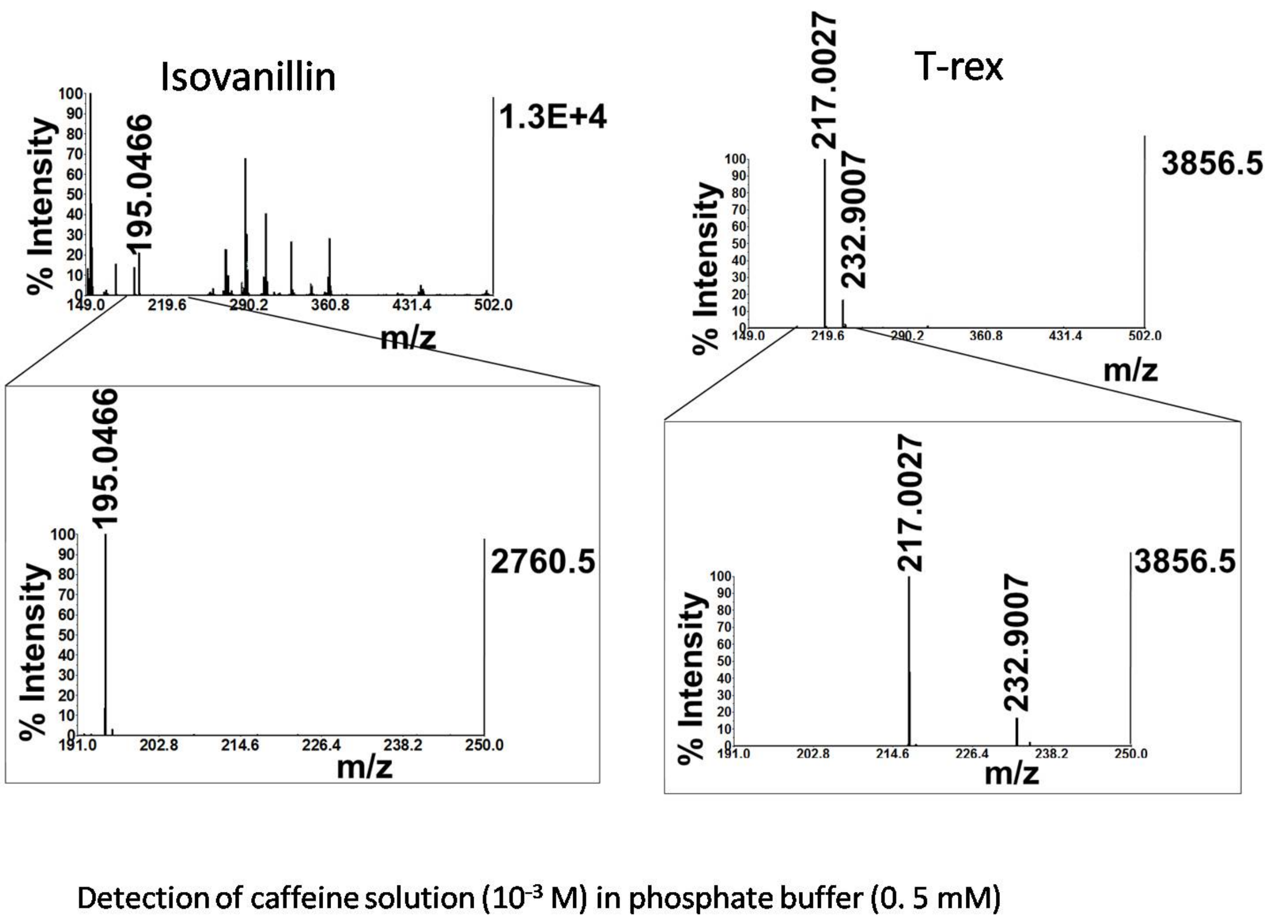

S3-A. Investigation of the SALDI/MS response of caffeine RaMassays (caffeine solution: 10^{-3} M) in phosphate buffer solutions (0.5 mM).

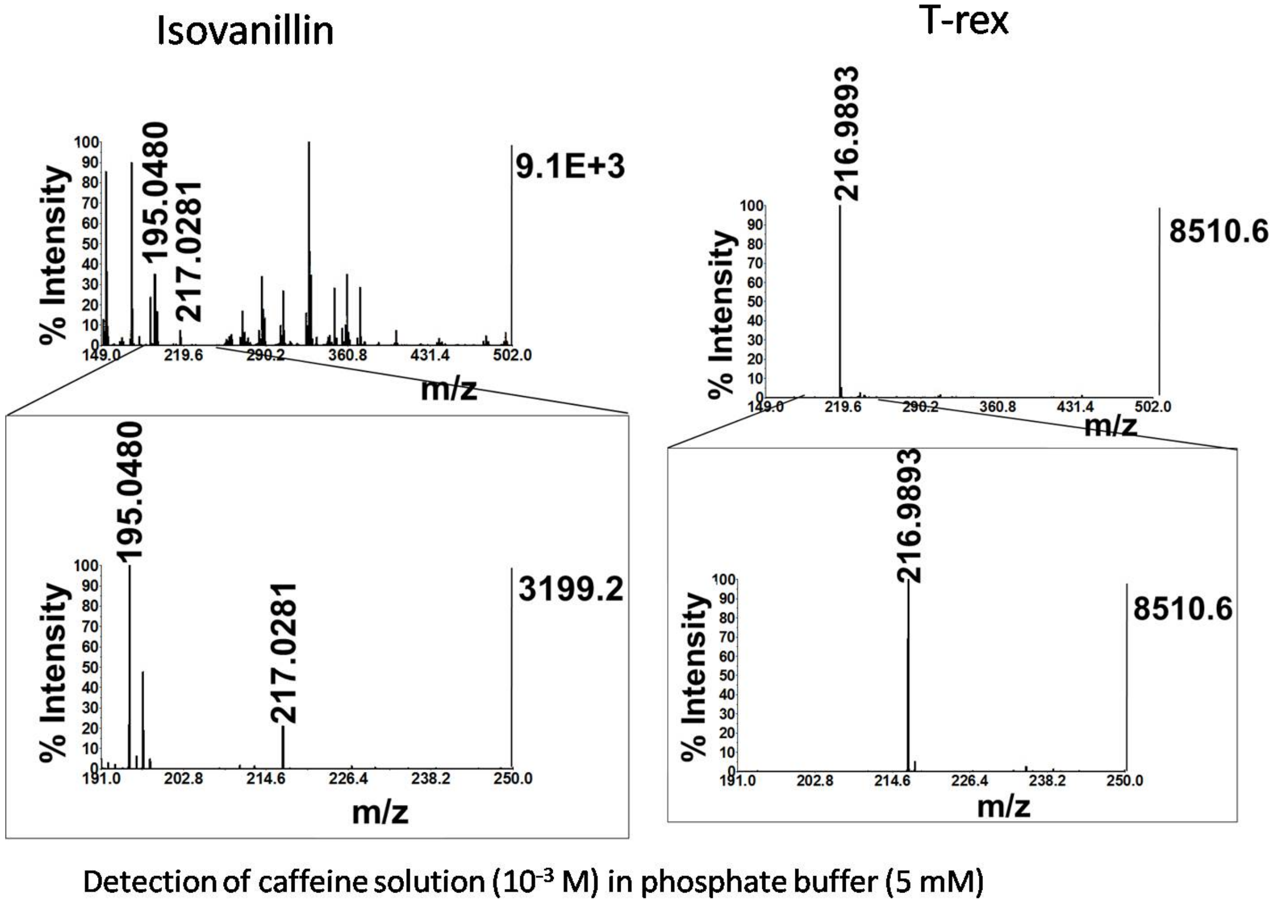

S3-B. Investigation of the SALDI/MS response of caffeine RaMassays (caffeine solution: 10^{-3} M) in phosphate buffer solutions (5 mM).

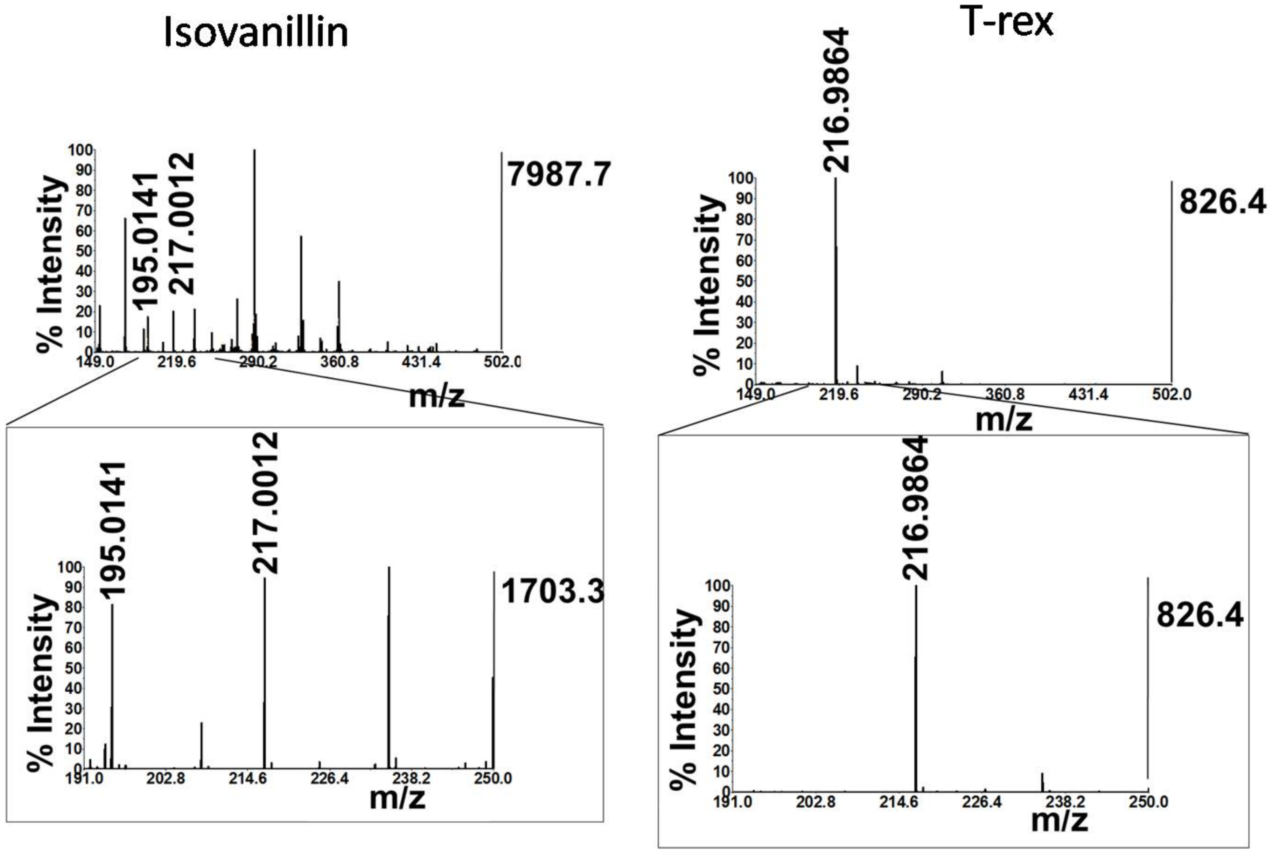

Detection of caffeine solution (10^{-3} M) in phosphate buffer (50 mM)

S3-C. Investigation of the SALDI/MS response of caffeine RaMassays (caffeine solution: 10^{-3} M) in phosphate buffer solutions (50 mM).
